# Combined Transarterial Embolization and Percutaneous CT-Guided Cryoablation of Renal Cell Carcinoma: A Retrospective Single-Center Study

**DOI:** 10.7759/cureus.116993

**Published:** 2026-09-27

**Authors:** Thomas J Vogl, Catharina Recoschewitz, Ruxandra Cojocaru, Andreea-Ioana Nica, Hamzah Adwan

**Affiliations:** 1 Radiology and Nuclear Medicine, Goethe University, University Hospital Frankfurt, Frankfurt, DEU

**Keywords:** embolization, image-guided ablation, interventional oncology, minimally invasive therapy, percutaneous cryoablation, renal cell carcinoma, thermal ablation

## Abstract

Purpose

The aim of this study was to evaluate the feasibility, safety, and oncological outcomes of combined transarterial embolization followed by percutaneous CT-guided cryoablation for renal cell carcinoma (RCC).

Material and methods

This retrospective single-center study included 24 patients with 24 biopsy-proven RCC who underwent transarterial embolization followed by percutaneous CT-guided cryoablation between May 2019 and August 2025. Procedural parameters, tumor characteristics, and oncological outcomes, as well as overall survival (OS), were assessed and analyzed.

Results

The mean tumor diameter was 3.9 ± 1.4 cm, and the mean ablation time was 26 ± 4.9 minutes. No major peri-procedural complications were observed. Early post-procedural imaging demonstrated initial complete ablation in 23 out of 24 tumors, equating to a rate of 95.8%. The rate of local tumor progression was 8.3% (2/24) and the rate of distant progression was 12.5% (3/24), which all occurred in patients without pre-existing metastatic disease. None of the patients with pre-existing metastatic disease developed local or distant disease progression during follow-up. The mean OS was 3.1 years (95% CI: 2.4-3.7) with a one- and two-year OS rate of 95% and 69.1%, respectively.

Conclusion

Combined transarterial embolization followed by percutaneous CT-guided cryoablation appears to be a safe and feasible treatment approach for selected patients with RCC, particularly those with limited surgical options.

## Introduction

Renal cell carcinoma (RCC) represents one of the most common malignancies of the kidney and constitutes a substantial global health burden [1]. In Germany, approximately 15,000 new cases are diagnosed each year [2]. Established risk factors include smoking, obesity, arterial hypertension, chronic kidney disease, and certain hereditary cancer syndromes [3].

Over recent years, the incidence of RCC has steadily increased, partly due to the widespread use of cross-sectional imaging modalities such as CT and MRI [4]. As a result, an increasing number of renal tumors are being detected incidentally at an early and localized stage [4]. These developments have led to a growing emphasis on nephron-sparing treatment strategies aimed at preserving renal function while maintaining oncological control [5].

Radical or partial nephrectomy remains the reference standard for the treatment of localized RCC due to its excellent long-term oncological outcomes [6]. However, surgical treatment is associated with perioperative morbidity and may not be suitable for all patients. For this reason, minimally invasive ablative techniques have emerged as alternative treatment approaches for selected patients with localized renal tumors [7].

Treatment options for RCC include radical or partial nephrectomy as well as image-guided ablative procedures such as radiofrequency ablation, microwave ablation, cryoablation, or stereotactic radiation [8]. While surgical resection remains the most frequently applied treatment modality, ablative techniques are most commonly used as second-line or alternative options, and their optimal role within the treatment algorithm continues to be evaluated [6].

Advances in cross-sectional imaging and interventional radiology have not only contributed to earlier tumor detection but have also enabled the development of minimally invasive image-guided treatment approaches [9]. Among these, percutaneous cryoablation allows for precise targeting of renal tumors under CT guidance with direct visualization of the ablation zone, offering a high degree of procedural control while minimizing damage to surrounding tissue [10].

According to current guidelines, cryoablation can be recommended as a treatment option, for example, in patients with increased surgical risk [11]. This technique is primarily recommended for small renal masses of <3-4 cm, while its role in larger tumors remains under investigation [6,11]. Cryoablation offers several potential advantages, including minimal invasiveness, organ preservation, short hospital stays, and a favorable safety profile. However, long-term oncological data remain limited, and recurrence rates may be higher compared to those following surgical treatment [12]. Consequently, the optimal role of cryoablation within the treatment algorithm-particularly in routine clinical practice and in patients with limited surgical options-remains under discussion.

Therefore, the aim of this retrospective single-center study was to evaluate the feasibility, safety, and oncological outcomes of combined transarterial embolization followed by percutaneous CT-guided cryoablation for RCC.

## Materials and methods

Study design

This retrospective single-center study was conducted according to the Declaration of Helsinki and was approved by the Ethics Committee of the Faculty of Medicine at Goethe University in Frankfurt (protocol code: 2025-2714). The requirement for informed consent was waived due to the retrospective design.

Patients who underwent percutaneous CT-guided cryoablation for RCC, aiming to achieve local tumor control, between May 2019 and August 2025 were included in this study. All patients received pre-treatment before cryoablation. Prior to cryoablation, all renal tumors included in this study were biopsy-proven RCC. Both endophytic and exophytic tumors were treated. All patients underwent preprocedural super-selective lipiodol-based transarterial embolization before CT-guided cryoablation as part of the institutional treatment protocol. The aim of the transarterial embolization procedure was to reduce tumor vascularity, improve tumor visualization, and facilitate safe and effective ablation, particularly in larger or anatomically complex tumors. Embolization was performed via transfemoral arterial access, and a total of 5-10 milliliters of lipiodol was applied. Embolization was carried out within two to four weeks prior to the cryoablation.

Patient selection 

Cryoablation was primarily performed in patients who were unsuitable for surgical resection, but also in patients with prior surgical treatments in whom cryoablation was considered a subsequent or salvage therapeutic option. Therefore, larger tumors were also treated in selected cases based on individual clinical decision-making, including for patients with limited therapeutic alternatives, palliative treatment intent, or explicit preference for the procedure, in whom surgical treatment was not feasible or was declined. Exclusion criteria were acute infection, coagulopathy, known allergies to anesthetic agents, and other contraindications to interventional procedures. 

Cryoablation 

All CT-guided cryoablation procedures were performed by the same interventional radiologist under local anesthesia using mepivacaine and sedation via diazepam, using the IceCure Medical ProSense™ system (IceCure Medical, Caesarea, Israel), which utilizes liquid nitrogen to generate the ice ball. Preprocedural planning was performed using MRI on 1.5-Tesla or 3-Tesla MAGNETOM scanners (Siemens Healthineers, Forchheim, Germany). 

Before probe insertion, the puncture site and probe trajectory were carefully planned based on a preprocedural planning CT scan. Tumor localization was assessed, and the optimal angle and depth of probe insertion were determined to ensure complete coverage of the tumor by the ice ball. A single probe (10 Gauge) was used for all ablation procedures. In the case of larger or oval-shaped tumors, intraprocedural repositioning of the probe was performed to achieve complete ablation.

The cryoprobe was advanced under CT guidance and positioned within the tumor according to the preprocedural planning. Throughout the procedure, repeated CT imaging was used to ensure correct probe placement and monitor the ablation procedure, including ice-ball formation and the distance to adjacent organs and the skin to minimize the risk of complications. Additionally, hydrodissection using normal saline was performed in 16 patients due to adjacent bowel structures to minimize the risk of thermal injury.

Cryoablation was performed following a freeze-thaw-freeze protocol, with freezing and thawing cycles lasting five to 10 min each. After completion of the second freeze cycle, the probe tip was warmed to allow for detachment from the ice ball and to ensure the cryoprobe could be safely removed. A patient case is shown in Figure 1.

**Figure 1:**
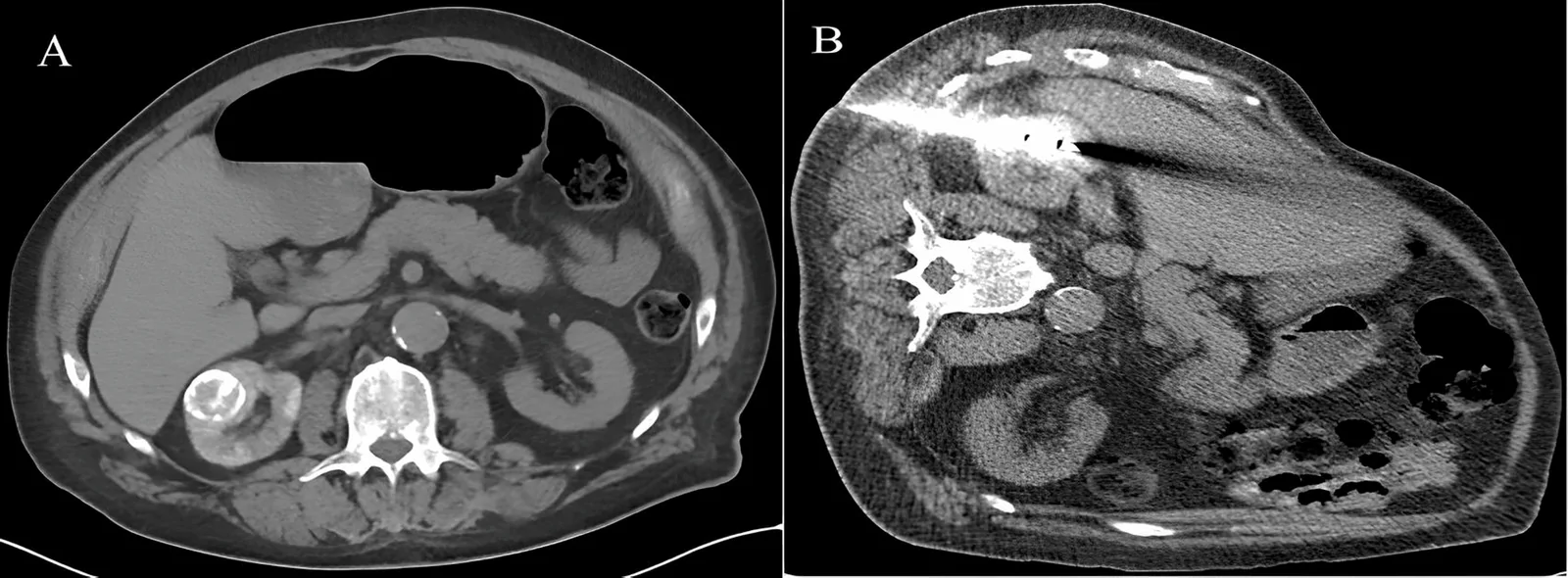
A 50 years-old patient with right-sided RCC. A: CT image after embolization of the tumor. B: During ablation with a positioned cryoprobe in the tumor.

Imaging follow-up and definitions 

Major complications were defined in accordance with the Society of Interventional Radiology (SIR) classification system [13]. Patients were discharged on the same treatment day if no complications occurred.

Complete ablation was defined as complete coverage of the tumor by the ablation zone with a sufficient ablative margin of 5 mm and the absence of residual contrast enhancement on first postinterventional MRI, indicating no evidence of viable residual tumor tissue, in accordance with standardized ablation reporting criteria proposed by Ahmed et al. [14]. Residual disease refers to persistent viable tumor tissue at the ablative margin identified on follow-up imaging, whereas local tumor progression was defined as newly appearing contrast-enhancing tumor tissue at the margin of the ablation zone after at least one follow-up study without evidence of residual viable tumor [14]. In case of residual disease, another session of cryoablation was performed. Distant disease progression was defined as the development of new tumor lesions outside the treated ablation zone, including new renal lesions in non-ablated areas and extrarenal metastatic lesions.

An MRI scan was performed 24 h post-ablation to confirm complete tumor ablation. After this initial post-interventional MRI, follow-up imaging using contrast-enhanced CT and MRI was routinely performed at three-month intervals to exclude renal tumor progression and distant metastasis. In case of renal tumor progression or distant metastasis, the cases were discussed interdisciplinarily to choose further appropriate treatment.

Statistical analyses

All statistical analyses were performed using SPSS® GradPack 27.0 (IBM Corp., Armonk, NY, USA). Continuous variables were summarized as means ± standard deviations. Follow-up time was additionally presented as a median. Categorical variables were presented as frequencies and percentages. Overall survival (OS) was defined as the time from cryoablation to death from any cause or last follow-up and estimated using the Kaplan-Meier method. 

## Results

Patient and tumor characteristics

A total of 24 patients with 24 RCCs who underwent cryoablation were evaluated in this study; 19 patients were male (79.2%), and five were female (20.8%), with a mean age of 68.4 ± 15.8 years. 

The mean tumor diameter was 3.9 ± 1.4 cm, corresponding to a mean tumor volume of 16.3 ± 17.1 cm3. Fourteen tumors (58.3%) measured ≤3 cm, while 10 tumors (41.7%) were larger than 3 cm. Three patients had metastatic disease at the time of treatment at a rate of 12.5% (3/24). The rate of patients with recurrent disease was also 12.5% (3/24). Table 1 summarizes the patient and tumor characteristics.

**Table 1:** Patient and tumor characteristics.

Parameter	Value
Number of patients	24
Female sex, n (%)	5 (20.8)
Male sex, n (%)	19 (79.2)
Mean age	68.4 ± 15.8 years
Patients with recurrent disease, n (%)	3 (12.5)
Patients with metastatic disease, n (%)	3 (12.5)
Number of tumors	24
Clear cell renal carcinoma, n (%)	21 (87.5)
Papillary renal cell carcinoma, n (%)	3 (12.5)
Mean tumor diameter	3.9 cm ± 1.4 cm
Mean tumor volume	16.3 ± 17.1 cm^3^
Tumor size	
≤3 cm, n (%)	14 (58.3)
>3 cm, n (%)	10 (41.7)

Procedural outcomes

A total of 25 cryoablation procedures were performed under CT guidance; one patient underwent a second procedure due to incomplete ablation. The mean ablation time was 26 ± 4.9 minutes, and the rate of initial complete ablation was 95.8% (23/24). No major peri-procedural complications were reported. Patients were positioned in the prone or lateral decubitus position depending on tumor location and the anticipated access route. Among the 25 cryoablation procedures, six were performed in the prone position, whereas the remaining procedures were carried out in the lateral decubitus position. Table 2 shows the details of the ablation procedures.

**Table 2:** Characteristics of ablation procedures.

Parameter	Value
Total number of ablations	25
Ablations in prone position, n (%)	6 (24)
Ablations in lateral decubitus position, n (%)	19 (76)
Mean ablation time	26 ± 4.9 minutes
Initial complete ablation, n (%)	23 (95.8)
Major complications, n (%)	0 (0.0)

Oncological outcomes and survival 

The rate of local tumor progression was 8.3% (2/24), and the rate of distant progression was 12.5% (3/24). The mean OS was 3.1 years (95% CI: 2.4-3.7), with one- and two-year OS rates of 95% and 69.1%, respectively (Figure 2). A total of three deaths were documented during a mean follow-up time of 1.4 ± 0.9 years (median follow-up time: one year). 

**Figure 2:**
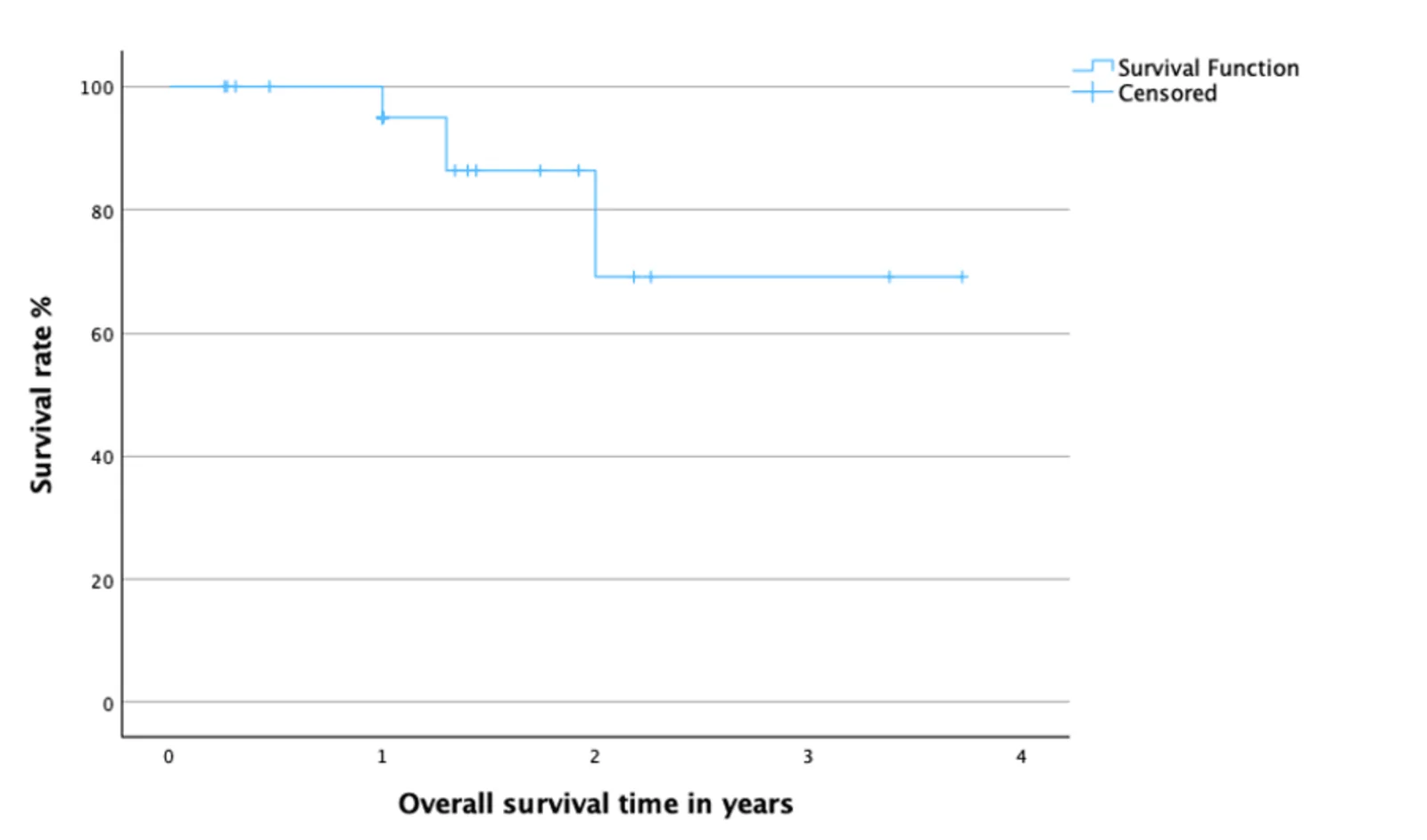
Kaplan–Meier curve of overall survival.

Subgroup analyses

Descriptive subgroup analyses were performed according to tumor size (≤3cm vs. >3cm) and metastatic status. No local tumor progression was observed in patients with tumors ≤3cm, whereas local tumor progression occurred in two patients with tumors >3cm. Distant progression was observed in one patient with a tumor ≤3cm and in two patients with a tumor larger than 3cm. One death occurred in the subgroup of patients with a tumor size ≤3cm, compared to two documented deaths in patients with tumors larger than 3cm. None of the patients with pre-existing metastatic disease developed local tumor progression or distant disease progression during follow-up. Among the 21 patients without pre-existing metastatic disease, local tumor progression occurred in two patients (9.5%), while distant disease progression occurred in three patients (14.3%). Due to the limited sample size, subgroup analyses were considered descriptive in nature. 

## Discussion

In this retrospective single-center cohort, combined transarterial embolization followed by percutaneous CT-guided cryoablation demonstrated favorable technical feasibility, safety, and reproducibility for the treatment of RCC across a heterogeneous patient population, including those with both small and larger tumors. Cryoablation was successfully performed even in patients with relevant comorbidities or limited surgical options, highlighting its clinical applicability. Cryoablation is well established for small, localized RCCs-most commonly tumors ≤3 cm (stage T1a)-and is recommended as an alternative to partial nephrectomy in the current guidelines of both the American Urological Association and the European Association of Urology [6,11].

A key strength of cryoablation observed in this study is its procedural simplicity and controllability. CT guidance allows for precise probe placement and direct visualization of ice ball formation, enabling accurate monitoring of the ablation margins throughout the procedure [10]. This high level of procedural control contributes to the excellent safety profile observed in our cohort and facilitates reliable lesion coverage without the need for procedural escalation or conversion. Importantly, no treatment-related complications occurred, underscoring the favorable procedural safety profile of the technique even in anatomically or clinically complex cases.

Our findings are consistent with previously published data reporting low complication rates for renal cryoablation [15]. In a larger cohort of 147 patients, Schaarschmidt et al. reported only a single treatment-related complication, supporting the finding that cryoablation is associated with a very low procedural risk [16]. The similarity between our results and those from larger series suggests that the favorable safety profile of cryoablation is not limited to highly selected populations but can also be achieved in real-world clinical practice.

While partial nephrectomy remains the standard of care for localized renal tumors [7], minimally invasive cryoablation represents a valuable alternative for selected patients, particularly those who are unsuitable for surgery due to comorbidities or prior renal interventions. In addition to its favorable safety profile, cryoablation offers several practical advantages, including organ preservation, repeatability, short hospital stays, and minimal invasiveness [7]. It can also be valuable for tumors in anatomically challenging locations. For example, endophytic renal tumors, which can be difficult to access surgically, are safely and effectively treated using image-guided cryoablation [17].

However, its role in larger renal masses remains under investigation. Importantly, all patients in our cohort underwent preprocedural transarterial embolization before cryoablation as part of the institutional treatment protocol. The rationale behind this was to reduce tumor vascularity and facilitate safe and effective ablation, particularly in larger or anatomically complex renal tumors. The preprocedural embolization may have contributed to procedural feasibility and local tumor control while enhancing safety by reducing tumor vascularity and improving visualization during cryoablation. Therefore, a potential influence of embolization on procedural and oncological outcomes cannot be fully excluded. However, cryoablation remained the primary local ablative treatment modality in all patients. 

Descriptive subgroup analyses also suggested less favorable oncological outcomes in tumors larger than 3 cm for our cohort, including higher rates of local recurrence and death. However, these findings should be interpreted cautiously due to the limited sample size and retrospective study design. Comparative studies evaluating tumors >3 cm have demonstrated higher rates of local recurrence and lower disease-free survival following cryoablation compared with partial nephrectomy, whereas metastasis-free and cancer-specific survival remained comparable between treatment approaches [18]. This is further supported by the meta-analysis by Gao et al., which included 2.011 patients from 10 studies, demonstrating significantly higher local recurrence rates after cryoablation compared with partial nephrectomy, while recurrence-free and overall survival remained comparable between treatment groups [12]. These findings are consistent with the broader literature reporting increased local recurrence after cryoablation, particularly for larger tumors, whereas oncological outcomes may remain comparable in selected patient populations despite limited long-term follow-up data [19,20]. The oncological findings of the present cohort are generally in line with previous published cryoablation series reporting favorable procedural safety and acceptable cancer-specific outcomes despite higher local recurrence rates compared with surgical treatment. However, direct comparison with larger surgical cohorts remains limited due to differences in patient selection, the inclusion of metastatic and previously treated patients, and the adjunctive use of preprocedural embolization in the present study. In addition to oncological considerations, previous studies have also demonstrated advantages of ablative therapies regarding renal function preservation. A meta-analysis by Wen et al. including 2.107 patients with solitary kidney tumors reported significantly lower rates of renal function decline and new-onset chronic kidney disease following ablative therapy compared with partial nephrectomy, despite higher local recurrence rates [21]. These findings highlight that treatment selection should not only consider oncological durability but also renal function preservation, procedural risk, patient comorbidities, and quality of life. 

 In addition, several studies have emphasized that patients undergoing cryoablation are often older and present with more comorbidities compared with surgical cohorts, which may substantially influence overall survival independent of the ablative technique itself [20]. While partial nephrectomy remains the standard of care, providing excellent long-term oncological control, it is associated with a higher perioperative risk of complications [22], especially in patients with relevant comorbidities or prior renal interventions. Cryoablation, by contrast, is a minimally invasive treatment option with a favorable safety profile, short procedural time, and low complication rates, as also demonstrated in the present study. Therefore, the choice between surgical and ablative treatment strategies should be considered on a case-by-case basis, and oncological durability should be balanced against procedural risk and patient-specific factors. 

Limitations

The limitations of this study must be acknowledged, including its retrospective design, limited sample size, and relatively short follow-up duration, as well as a heterogeneous patient cohort. The relatively short follow-up duration further limits the assessment of long-term oncological control, recurrence patterns, and interpretation of overall survival estimates. The inclusion of patients with metastatic disease, prior renal interventions, and additional systemic therapies further limits direct interpretation of the oncological outcomes. Due to the retrospective study design and heterogeneous multimodal treatment pathways, complete tumor characterizations, including exact tumor-node-metastasis (TNM) stage, nephrometry scores, and standardized classification of primary versus recurrent disease, were not consistently available. Additionally, as all patients received adjunctive preprocedural embolization prior to cryoablation, a potential influence of embolization on the procedural and oncological outcomes cannot be fully excluded. We performed subgroup analyses; however, due to the limited number of patients and events, including deaths, a Cox regression analysis was not considered statistically reliable. Furthermore, the interpretation of overall survival estimates remains limited due to the relatively short and heterogeneous follow-up duration as well as the low number of death events within the study cohort. Nevertheless, our findings support the feasibility of this combined treatment approach in selected patients with limited therapeutic alternatives.

Future studies should aim to include longer follow-up periods to allow for a better assessment of long-term oncological outcomes and recurrence patterns as well as renal function. In addition, further investigations to identify tumor-specific factors that may influence treatment response and patient outcomes are warranted.

## Conclusions

The combination of transarterial embolization followed by percutaneous CT-guided cryoablation appears to be a safe and feasible minimally invasive treatment approach for selected patients with RCC, particularly those with limited surgical options. However, the present findings should be considered preliminary and interpreted within the limitations of the retrospective study design, small sample size, heterogeneous patient population, and relatively short follow-up duration. Further studies with larger cohorts and longer follow-up are warranted to determine the oncological efficacy and appropriate clinical role of this combined treatment approach.
